# First Report of a Thioredoxin Homologue in Jellyfish: Molecular Cloning, Expression and Antioxidant Activity of CcTrx1 from *Cyanea capillata*

**DOI:** 10.1371/journal.pone.0097509

**Published:** 2014-05-13

**Authors:** Zengliang Ruan, Guoyan Liu, Yufeng Guo, Yonghong Zhou, Qianqian Wang, Yinlong Chang, Beilei Wang, Jiemin Zheng, Liming Zhang

**Affiliations:** Department of Marine Biotechnology, Faculty of Naval Medicine, Second Military Medical University, Shanghai, China; Russian Academy of Sciences, Institute for Biological Instrumentation, Russian Federation

## Abstract

Thioredoxins (Trx proteins) are a family of small, highly-conserved and ubiquitous proteins that play significant roles in the resistance of oxidative damage. In this study, a homologue of Trx was identified from the cDNA library of tentacle of the jellyfish *Cyanea capillata* and named CcTrx1. The full-length cDNA of CcTrx1 was 479 bp with a 312 bp open reading frame encoding 104 amino acids. Bioinformatics analysis revealed that the putative CcTrx1 protein harbored the evolutionarily-conserved Trx active site ^31^CGPC^34^ and shared a high similarity with Trx1 proteins from other organisms analyzed, indicating that CcTrx1 is a new member of Trx1 sub-family. CcTrx1 mRNA was found to be constitutively expressed in tentacle, umbrella, oral arm and gonad, indicating a general role of CcTrx1 protein in various physiological processes. The recombinant CcTrx1 (rCcTrx1) protein was expressed in *Escherichia coli* BL21 (DE3), and then purified by affinity chromatography. The rCcTrx1 protein was demonstrated to possess the expected redox activity in enzymatic analysis and protection against oxidative damage of supercoiled DNA. These results indicate that CcTrx1 may function as an important antioxidant in *C. capillata*. To our knowledge, this is the first Trx protein characterized from jellyfish species.

## Introduction

Jellyfish (Scyphozoa), a class of Cnidaria, are abundant in pelagic oceanic waters. As a representative of macroplankton, they are continuously exposed to high light, solar radiation, as well as other deleterious exogenous factors such as microorganisms, pathogens and varying conditions of temperature, pressure and salinity, which may result in an overproduction of reactive oxygen species (ROS) [1]. Excessive ROS in cells can cause oxidative damages to DNA, lipid membranes and proteins. Intracellular redox homeostasis can also be disrupted by a subtle increase in the level of ROS [1], [2]. However, jellyfish have well adapted and presented tolerance to such harsh environmental conditions. Hence they are believed to possess robust mechanisms for eliminating ROS and maintaining the intracellular environment in a reduced state. Scientists have found that some proteins isolated from jellyfish have a strong radical-scavenging activity and reducing power [3]–[5]. Previously, we have reported the first peroxiredoxin (Prx) gene isolated from jellyfish species which showed the ability to reduce hydrogen peroxide and protect supercoiled DNA from oxidative damage [6]. However, until now, other important antioxidants, including thioredoxin, catalase, glutathione peroxidase and superoxide dismutase, have not yet been reported in jellyfish.

The thioredoxin system, comprising thioredoxin (Trx protein), thioredoxin reductase (TrxR) and NADPH, is a key antioxidant system in the defense against oxidative stress through its disulfide reductase activity [7]. Trx is a small protein which is evolutionarily conserved from prokaryotes to eukaryotes [8]–[10]. It relies on the Cys residues in its active site (Cys-Gly-Pro-Cys) for reduction of the protein disulfide bond [11], [12]. So far, three distinct forms of Trx have been cloned and characterized in various organisms. The classical cytosolic Trx (Trx1) with a molecular mass around 12 kDa is the one that has been most studied. It is mainly accumulated in the cytosol but can be translocated into the nucleus and secreted out of the cell under certain circumstances [13], [14]. Trx2 is a mitochondrial protein which has a special N-terminal mitochondrial translocation signal [15]. The third isoform, SpTrx, is a variant that is only expressed in spermatozoa [16]. Trx proteins have been demonstrated to play an important role in the resistance against oxidative stress and regulation of cellular redox homeostasis [17]. Moreover, Trx proteins have also been shown to perform a variety of biological functions, such as the elimination of free radicals, regeneration of proteins inactivated by oxidative stress, regulation of gene expression, protection against inflammation and control of apoptosis [18]–[21].

So far, a variety of Trx proteins have been identified and functionally characterized in organisms ranging from invertebrate to human [22], [23]. In Cnidaria, Trx sequences from hydras *Hydra magnipapillata* and *Hydra vulgaris* and sea anemone *Nematostella vectensis* have been deposited in the GenBank (accession number: XP002157650, XP_002159164 and XP_001638202, respectively). However, to our knowledge, no information is yet available for Trx in jellyfish species. Therefore, the importance of Trx in organism homeostasis and cellular oxidative defense mechanism triggered our interests to characterize the Trx gene in jellyfish and to gain a deeper insight into its biological activity.

*Cyanea capillata*, with a worldwide distribution, is one of the most common kinds of jellyfish in the Southeast China Sea. In this study, for the first, we cloned and characterized a full-length Trx cDNA, named as CcTrx1, from *C. capillata*. The tissue expression profile of CcTrx1 was studied, and the recombinant CcTrx1 (rCcTrx1) protein was expressed and purified. The disulfide reduction ability and antioxidant activity of rCcTrx1 protein were further investigated to elucidate its function as a reductive agent and role in the resistance of oxidative damage in *C. capillata*.

## Materials and Methods

### cDNA library construction and EST analysis

As we described in details previously [6], total RNA was extracted from the tentacle of *C. capillata* with Trizol Reagent (Invitrogen, Carlsbad, CA, USA), and the cDNA library was constructed using the SMART cDNA Library Construction Kit (Clontech, Mountain View, CA, USA) according to the manufacturer's instructions.

Based on BLASTx analysis of the EST sequences from the cDNA library, we discovered a Trx1 homologue. Thus this EST sequence, designated as CcTrx1 (*C. capillata* Trx1), was selected for further analysis. Complete sequencing of both strands of CcTrx1 cDNA was then carried out using an ABI Prism@ 3730 sequencer by a commercial sequencing company (Beijing Liuhe Huada Genomics Technology Co., Ltd.) to confirm it as a full-length cDNA.

### Sequence characterization of CcTrx1

The CcTrx1 cDNA and its deduced amino acid sequence were analyzed using appropriate bioinformatics tools. The homology search of CcTrx1 nucleotide sequence was conducted with the BLASTx algorithm (http://www.ncbi.nlm.gov/blast) [24]. The open reading frame (ORF) was determined using the ORF Finder program (http://www.ncbi.nlm.nih.gov/gorf/gorf.html). The presence of conserved domains was analyzed by using the InterProScan (http://www.ebi.ac.uk/Tools/pfa/iprscan/) and CDD (http://www.ncbi.nlm.nih.gov/Structure/cdd/cdd.shtml) programs [25], [26]. Multiple alignment was performed with the ClustalW2 program (http://www.ebi.ac.uk/Tools/msa/clustalw2/). The presence and location of signal peptide in the deduced amino acid sequence of CcTrx1 was predicted using the SignalP 4.1 Server (http://www.cbs.dtu.dk/services/SignalP/) [27]. A phylogenetic neighbor-joining (NJ) tree was constructed using the MEGA 4 software package with 2,000 bootstrap replicates. The molecular weight (MW) and theoretical isoelectric point (pI) were determined using the ProtParam tool (http://web.expasy.org/protparam/). A three dimensional model of the CcTrx1 protein was made using the SWISS-MODEL algorithm (http://swissmodel.expasy.org/) [28], and the model was viewed and modified by using PyMOL program (version 0.99rc6 for Windows) [29].

### Tissues distribution of CcTrx1 mRNA

Total RNA was extracted from 1 g of fresh tissues (tentacle, oral arm, umbrella and gonad, respectively) using UNIQ-10 Kit (Sangon Biotech, Shanghai, China) according to the manufacturer's instructions. The concentration of RNA was determined by a BioPhotometer (Eppendorf, Hamburg, Germany). Following the manufacturer's instructions of the PrimeScript RT Reagent Kit (TaKaRa, Otsu, Shiga, Japan), first strand cDNA was synthesized with the total RNA as template. Two primers employed for quantitative real-time PCR (qRT-PCR), qPCR-CcTrx1-F and qPCR-CcTrx1-R (Table 1), were designed to amplify a product of 80 bp. The *C. capillata* GAPDH gene (GenBank accession number KF595154) was used as an internal control in the reaction and amplified with the specific primers qPCR-CcGAPDH-F and qPCR-CcGAPDH-R (Table 1) that produced a fragment of 122 bp. As described previously [6], qRT-PCR was performed in a total volume of 25 µL using an ABI PRISM 7300 Sequence Detector (Applied Biosystems, Foster City, CA, USA). The reaction was performed with 40 cycles of programmed temperature control of 95°C for 15 s and 60°C for 31 s with a 30 s preheat at 95°C. Dissociation curve analysis was performed by gradual heating of the PCR products from 60 to 95°C at the end of each PCR reaction to confirm that the amplifications were specific. Relative gene expression was analyzed by the comparative Ct method (2^−ΔΔCt^ method) and the results were presented as the relative quantity values [30]. Ct values of CcTrx1 gene were normalized based on those for the GAPDH gene. All treatments were performed in triplicate, and data were presented as mean ± SE (n = 3). The significance of the differences of tissue-specific expression of CcTrx1 between tentacle and other tissues was analyzed with one-way analysis of variance (ANOVA) and *P* values lower than 0.05 were considered statistically significant. Statistical analysis were carried out using IBM SPSS Statistics 19.

**Table 1:** Primers used for cloning and qPCR in this study.

Primer name	Nucleotide sequence (5′ →3′)
qPCR-CcTrx1-F	GGAGTTTTCCAATACCTATGGTGAT
qPCR-CcTrx1-R	CACAGGCTTCTGATGTGTCAGT
qPCR-CcGAPDH-F	GGTGCCCATCAAAACATTATC
qPCR-CcGAPDH-R	GACACATCAGCAACTGGAACAC
rCcTrx1-F	GCGGGAATTCCATATGGTTAGAGA
rCcTrx1-R	CCGCTCGAGTTTATGACTCTTAATC

### CcTrx1/pET-24a recombinant plasmid construction

The coding region of CcTrx1 cDNA was amplified from the cDNA library using standard PCR with the primers rCcTrx1-F and rCcTrx1-R (Table 1). The primers were designed to incorporate *Nde* I and *Xho* I restriction enzyme sites (underlined in Table 1), respectively. The digested PCR products and pET-24a vector (Novagen, Madison, WI, USA) were ligated at room temperature (25°C) for 1 hour using T4 DNA ligase (NEB, Ipswich, MA, USA). The ligation mixture was then transformed into *E. coli* TOP 10 competent cells (BioMed, Beijing, China). Successfully transformed cells were identified using the agar plates containing 100 µg/mL kanamycin, followed by nucleotide sequencing of both strands through outsourcing service provided by Beijing Liuhe Huada Genomics Technology Co., Ltd., to confirm in-frame insertion. This company was using an automatic DNA sequencer (ABI Prism@ 3730, Applied Biosystems, USA).

### Expression and purification of the rCcTrx1 protein

The recombinant plasmid was transformed into the *E. coli* BL21 (DE3) strain for protein expression as described in our previous study with minor modifications [6]. Protein expression was induced with a final concentration of 1 mM isopropyl-β-D-thiogalactoside (IPTG) and bacteria were harvested after incubation for 8 hours at 25°C with shaking at 150 rpm. Subsequently, the bacteria were centrifuged at 12,000×*g* for 6 min, and the pellets were resuspended in binding buffer containing 20 mM NaH_2_PO_4_, 500 mM NaCl and 30 mM imidazole (pH 7.4). The resuspended bacterial pellets were sonicated for 6 min in an ice bath and the lysate was centrifuged again at 12,000×*g* for 30 min at 4°C. The supernatant incorporated the His-tagged recombinant CcTrx1 protein was collected and applied to a 5 mL HisTrap™ HP metal affinity column in the ÄKTA protein purification system (GE Healthcare, Milwaukee, WI, USA) at a flow rate of 1 mL/min, monitored by measuring the absorbance at a wavelength of 280 nm. After washing the column with 100 mL of binding buffer (20 mM NaH2PO4, 500 mM NaCl, 30 mM imidazole, pH 7.4), the recombinant CcTrx1 protein was eluted from the column by the addition of elution buffer (20 mM NaH2PO4, 500 mM NaCl, 500 mM imidazole, pH 7.4). Protein samples collected from different steps of the purification were analyzed via 12% (*w*/*v*) sodium dodecyl sulfate-polyacrylamide gel electrophoresis (SDS-PAGE) and the gel was stained with Coomassie blue R-250 (Beyotime, Haimen, Jiangsu, China) [31]. Protein concentrations were measured by Bradford assay using bovine serum albumin (BSA) as standard. [32].

### Western blot analysis

Western blot analysis was performed as described previously with minor modification [6]. Briefly, the separated proteins were analyzed by SDS-PAGE and then electroblotted onto a polyvinylidene difluoride membrane (Millipore, Billerica, MA, USA). Subsequently, the membrane was blocked with 5% (*w/v*) fat-free milk powder in tris-buffered saline (TBST) containing 50 mM Tris, 150 mM NaCl, 0.05% (*v/v*) Tween 20 (pH 7.6). Anti-His antibody was then incubated with membrane at 4°C overnight after washing the membrane with TBST. Subsequently, HRP-labeled goat anti-mouse IgG (Beyotime, Haimen, Jiangsu, China) was used as the secondary antibody. The chemiluminescent detection of cross-reacting proteins was performed by the G:BOX system (Syngene, Cambridge, UK).

### Insulin disulfide reduction assay

Trx protein catalyzes the reduction of the two inter-chain disulfide bonds of insulin in the presence of DTT, and a white precipitate is formed mainly from the free B chain of insulin which is insoluble and could be monitored by measuring absorbance at 650 nm [33], [34]. Hence, a turbidimetric assay was developed to measure the disulfide reducing activity of the purified rCcTrx1 by recording the rate of the precipitation of the free insulin chain B. Briefly, the reaction mixture contained a final volume of 1 mL with 100 mM phosphate buffered saline (PBS), 1.25 mg/mL bovine insulin (Sigma, USA), 2 mM ethylenediamine tetraacetic acid (EDTA) and 8 µg/mL purified rCcTrx1 protein or the heat-inactivated purified rCcTrx1 protein as negative control. The reaction was initiated by adding 2 µL of 1 M DTT and monitored by measuring absorbance at 650 nm, and a group of 8 µg/mL purified rCcTrx1 protein without adding DTT was used as another negative control. The rate of precipitation was calculated as the increase ΔA_650_× min^−1^ in the interval between 0 and 1.0. The specific activity was calculated as ΔA_650_× min^−1^× 1000/µg of rCcTrx1 protein [33].

### DNA cleavage assay by the metal-catalyzed oxidation system

In order to assess the ability of the rCcTrx1 protein to protect supercoiled DNA from oxidative damage, a DNA cleavage assay was performed by the metal-catalyzed oxidation (MCO) assay according to the method described previously with slight modifications [6], [35]. Briefly, the reaction was conducted at 37°C for 2 h in 50 µL reaction systems contained 1 µg pET-24a supercoiled plasmid DNA, 35 µM FeCl_3_, 10 mM DTT and increasing concentrations of rCcTrx1 protein ranging from 25 to 200 µg/mL in 50 mM 4-(2-hydroxyethyl)-1-piperazineethanesulphonic acid (HEPES) (pH 7.0). DNA protection effect was evaluated by 1% (*w/v*) agarose gel electrophoresis and stained with Golden View (BioMed, Beijing, China).

## Results

### Sequence characterization of CcTrx1

**Figure 1:**
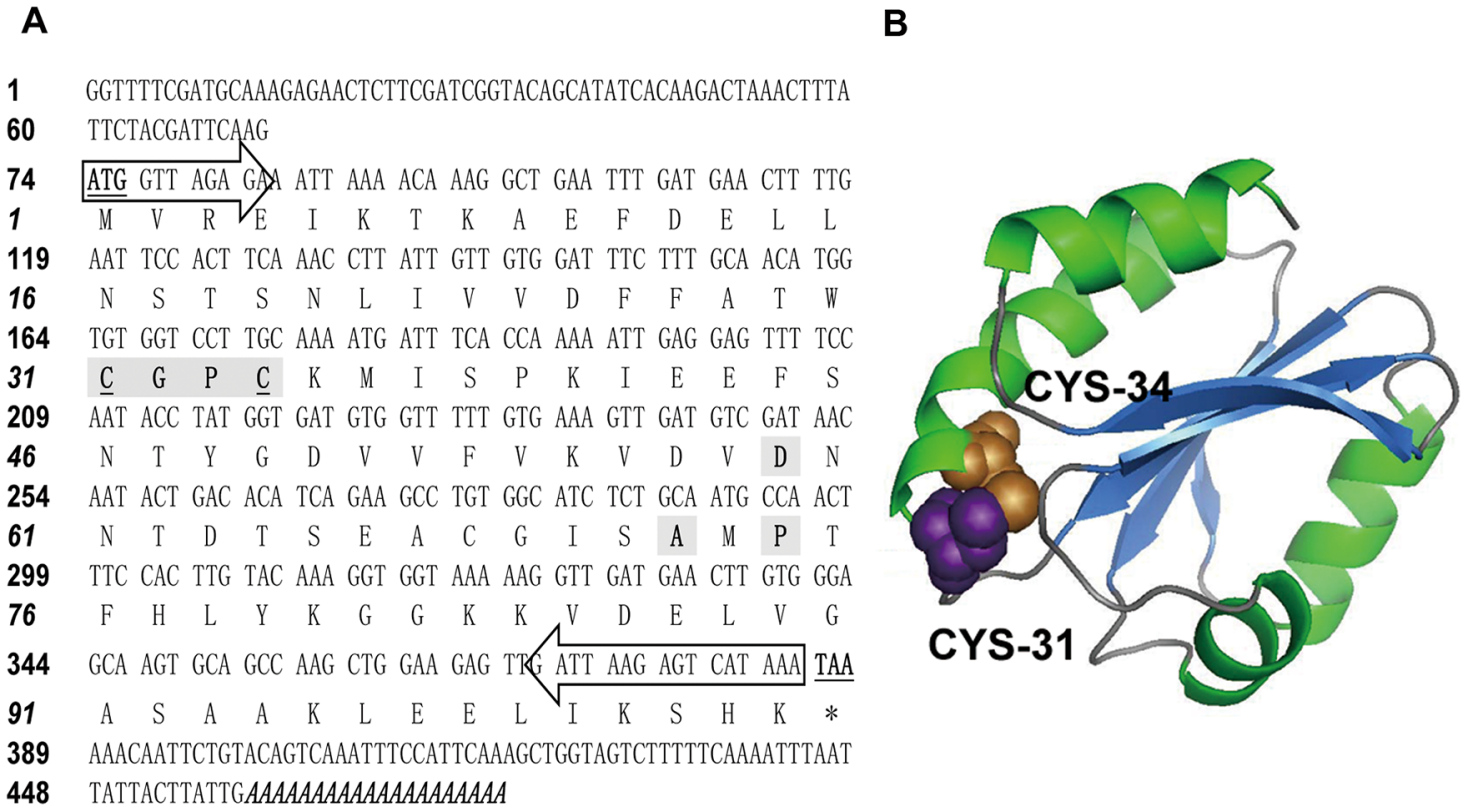
Sequence analysis of CcTrx1. (A) The full-length cDNA nucleotide and deduced amino acid sequences of CcTrx1. This cDNA nucleotide sequence has been deposited in the GenBank under accession number KF201510. The start (ATG) and stop (TAA) codons are bold and underlined. The characteristic active site (^31^CGPC^34^) is bold and shaded with the active cysteine residues are underlined. The conserved aspartic acid at position 59, alanine at position 72 and proline at position 74 are bold and shaded. The poly (A) tail is shown bold and italicized at the end of sequence. The locations of primers used for cloning and expression of the recombinant protein are indicated with arrows. (B) The predicted three-dimensional structure of the CcTrx1 protein. Alpha-helices are shown in green, β-strands in blue, and β-turns in grey. Balls in purple and copper represent CYS^31^ and CYS^34^, respectively.

The full-length cDNA sequence of CcTrx1 was isolated from a cDNA library of tentacle of *C. capillata* and deposited in the GenBank under accession No. KF201510. As shown in Figure 1A, the full-length CcTrx1 cDNA was composed of 479 bp containing a 73 bp 5′-untranslated region (UTR), a single open reading frame (ORF) of 312 bp and a 94 bp 3′-UTR including a stop codon (TAA) and a poly (A) tail. The ORF encoded a putative protein of 104 amino acids with a calculated molecular mass of 11.5 kDa and an estimated pI of 5.1. The characteristic Trx redox active site ^31^CGPC^34^ was also well conserved in CcTrx1 [23], [36]. In addition, no signal peptide could be predicted within the deduced amino acid sequence of CcTrx1, indicating that it might be located in the cytosol.

The tertiary structure of the CcTrx1 protein was also predicted using the SWISS-MODEL programs with the Trx1 protein from *Homo sapiens* (PDB ID: 2IIY, chain A) as the template. As shown in Figure 1B, the CcTrx1 protein consisted of five β-strands, composed of a central core, and four α-helices. The conserved ^31^CGPC^34^ redox-active site was located after the second β-strand and followed by the second α-helix. This structure was very similar to those of Trx1 proteins from Chinese mitten crab *Eriocheir sinensis*, colibacillus *E. coli* and Pacific white shrimp *Litopenaeus vannamei* [37]–[39].

### Homology and phylogenetic analysis of CcTrx1

**Figure 2:**
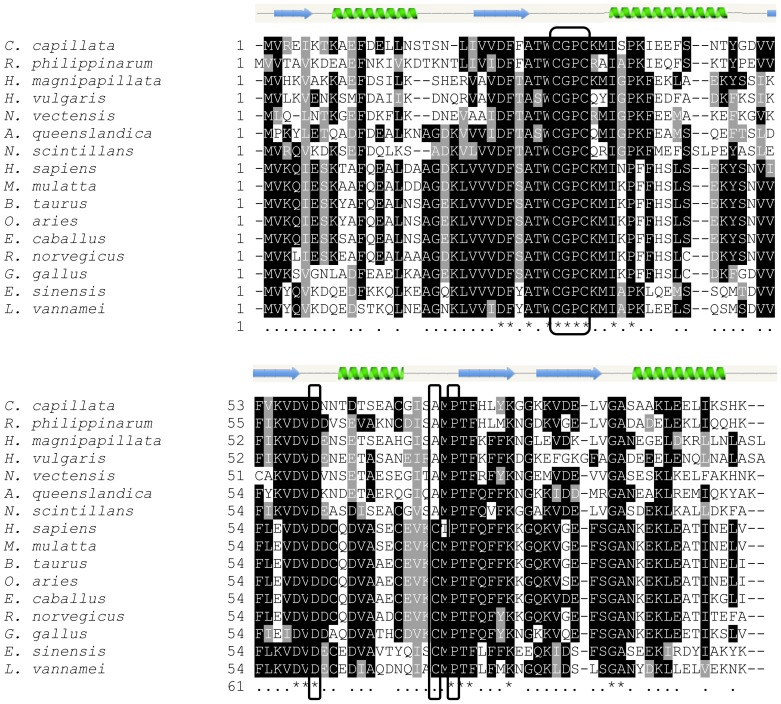
Multiple sequence alignment of the CcTrx1 protein with identified Trx1 proteins from various species. The completely conserved residues across all the aligned sequences are highlighted with a shade of black and an asterisk (*) on the bottom. Meanwhile, the highly conserved residues are indicated by dots (.) and shaded in gray, and the absent amino acids are indicated by dashes (−). The characteristic CGPC active site, the conserved Asp60, Cys73 and the cis-proline residue are boxed. Secondary structure of CcTrx1 is shown as labeled green spirals (α helices) and blue arrows (β strands).

Multiple sequence alignment illustrated that the predicted amino acid sequence of the CcTrx1 protein displayed a significant homology with other identified Trx1 proteins from various species (Figure 2). Because of the sequence similarity, we speculated that CcTrx1 might belong to the cytosolic Trx1 sub-family. Multiple sequence alignment also revealed that the characteristic active site CGPC was highly conserved in all the Trx1 proteins analyzed. An Asp60 residue, previously reported to control the pH dependent dimerization [40], was also well conserved in the CcTrx1 protein. However, Cys73 residue, reported to contribute to the formation of the Trx dimer [41], was not found in the CcTrx1 protein, and the corresponding residue was alanine that was also found in the Trx1 proteins from *H. magnipapillata, H. vulgaris*, *N. vectensis*, *Amphimedon queenslandica*, *Ruditapes philippinarum* and *Noctiluca scintillans*. In addition, The CcTrx1 protein possesses the characteristic cis-proline residue at position 74 which had been demonstrated to play a significant role in stability of Trx1 proteins [42], [43].

**Figure 3:**
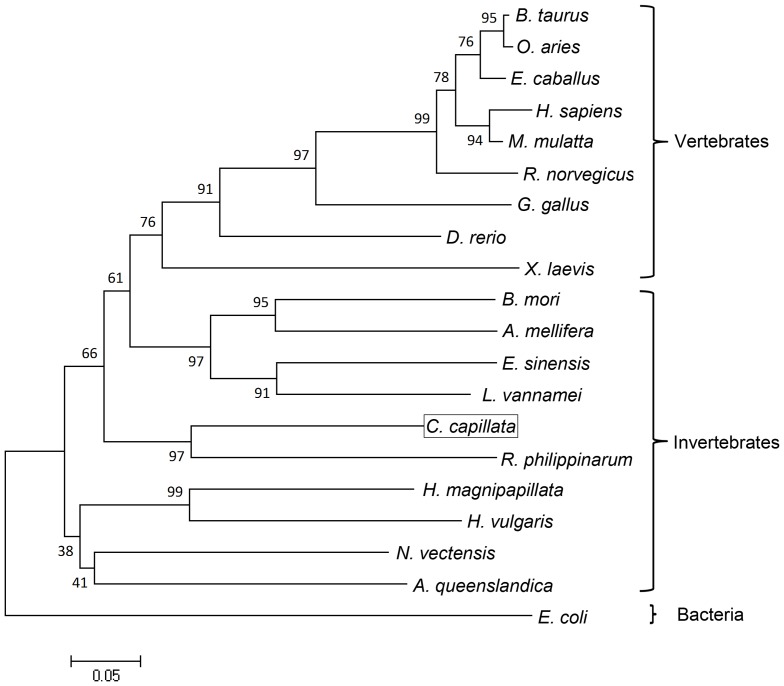
Phylogenetic analysis of the CcTrx1 protein compared with other known Trx1 proteins. The numbers on the nodes indicate percentage frequencies in 2000 bootstrap replications. The amino acid sequence from *E. coli* was used as the out-group. The common names, sizes as well as GenBank accession numbers of the selected Trx1 amino acid sequences are indicated in Table 2.

Pairwise comparisons revealed that the CcTrx1 protein shared 56.0–79.2% similarities with Trx1 proteins from other organisms, including vertebrates (human *H. sapiens*, rhesus monkey *Macaca mulatta*, cattle *Bos taurus*, sheep *Ovis aries*, pig *Sus scrofa*, horse *Equus caballus*, Norway rat *Rattus norvegicus*, chicken *Gallus gallus* and zebrafish *Danio rerio*) and invertebrates (Chinese mitten crab *E. sinensis*, Pacific white shrimp *L. vannamei*, hydras *H. magnipapillata* and *H. vulgaris*, sea anemone *N. vectensis*, sponge *A. queenslandica*, clam *R. philippinarum*, silkworm *Bombyx mori* and honeybee *Apis mellifera*). The CcTrx1 protein was also similar to that from algae, such as algae *N. scintillans* (Table 2). Among these species, the CcTrx1 protein had the highest similarity with that from *R. philippinarum*. A phylogenetic tree was also constructed based on the deduced amino acid sequence of the CcTrx1 protein and the Trx1 proteins of 19 representative species obtained from GenBank database (Figure 3). In the tree, the vertebrates were clustered distinctly. CcTrx1 was positioned into a branch of the invertebrate sub-cluster and most closely resembled the Trx1 from *R. philippinarum*, which was in agreement with the result obtained from pairwise alignment. This grouping was well-supported by bootstrapping.

**Table 2:** The deduced amino acid sequence of the CcTrx1 protein compared with the Trx1 proteins from other species.

Species name	Common name	Accession number	Sequence size (aa)	Identity (%)	Similarity (%)
*Cyanea capillata*	Jellyfish	KF201510	104	-	-
*Homo sapiens*	Human	AAF86466	105	44.0	62.4
*Macaca mulatta*	Rhesus monkey	AAA36921	105	46.8	62.4
*Bos taurus*	Cattle	NP_776393	105	48.6	61.5
*Ovis aries*	Sheep	NP_001009421	105	48.6	61.5
*Sus scrofa*	Pig	NP_999478	105	48.6	61.5
*Equus caballus*	Horse	NP_001075282	105	50.5	64.8
*Rattus norvegicus*	Norway rat	NP_446252	105	46.7	61.0
*Gallus gallus*	Chicken	NP_990784	105	49.5	63.8
*Xenopus laevis*	African clawed frog	NP_001088487	105	41.9	61.0
*Eriocheir sinensis*	Chinese mitten crab	ACQ59118	105	46.7	65.7
*Litopenaeus vannamei*	Pacific white shrimp	ACA60746	105	49.5	69.5
*Danio rerio*	Zebrafish	NP_001002461	107	56.1	67.3
*Bombyx mori*	Silkworm	NP_001091804	106	45.3	58.5
*Apis mellifera*	Honey bee	XP_392963	105	45.7	61.0
*Ruditapes philippinarum*	Clam	AET44428	106	59.4	79.2
*Noctiluca scintillans*	Algae	ABV22334	105	57.5	72.6
*Hydra magnipapillata*	Hydra	XP002157650	105	50.9	68.9
*Hydra vulgaris*	Hydra	XP_002159164	106	38.3	59.8
*Nematostella vectensis*	Sea anemone	XP_001638202	103	49.5	67.6
*Amphimedon queenslandica*	Sponge	XP_003383163	106	48.1	69.8
*Escherichia coli*	Colibacillus	P0AA25	109	35.8	56.0

The accession numbers are from the GenBank database.

### Tissue-specific expression of CcTrx1

**Figure 4:**
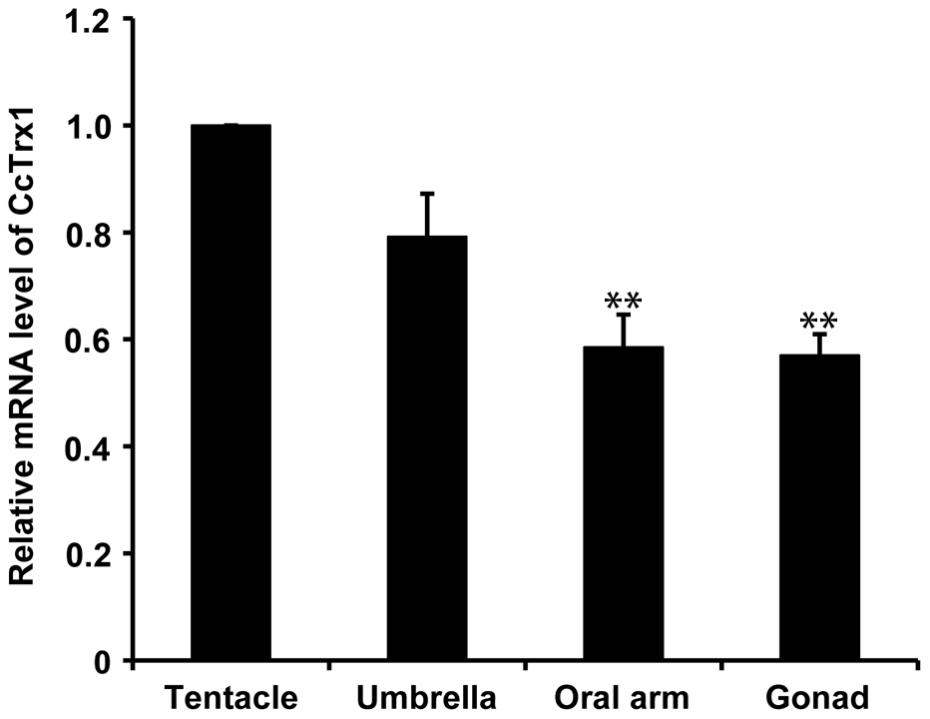
qRT-PCR analysis of CcTrx1 tissue-specific expression. Relative expression was calculated using the 2^−ΔΔCt^ method with GAPDH as the reference gene. The results are presented as the relative quantity values. All treatments were performed in triplicate, and data were presented as mean ± SE (n = 3, ** *P*<0.01 *vs*. tentacle).

As shown in Figure 4, the CcTrx1 mRNA was mainly expressed in the tentacle and umbrella with no significant differences between them, but was significantly lower in the oral arm and gonad (*P*<0.01 *vs*. tentacle). Moreover, for both of CcTrx1 and GAPDH genes, there was only one peak in the dissociation curve analysis, indicating that the PCR products were specifically amplified (data not shown).

### Expression and purification of the rCcTrx1 protein

**Figure 5:**
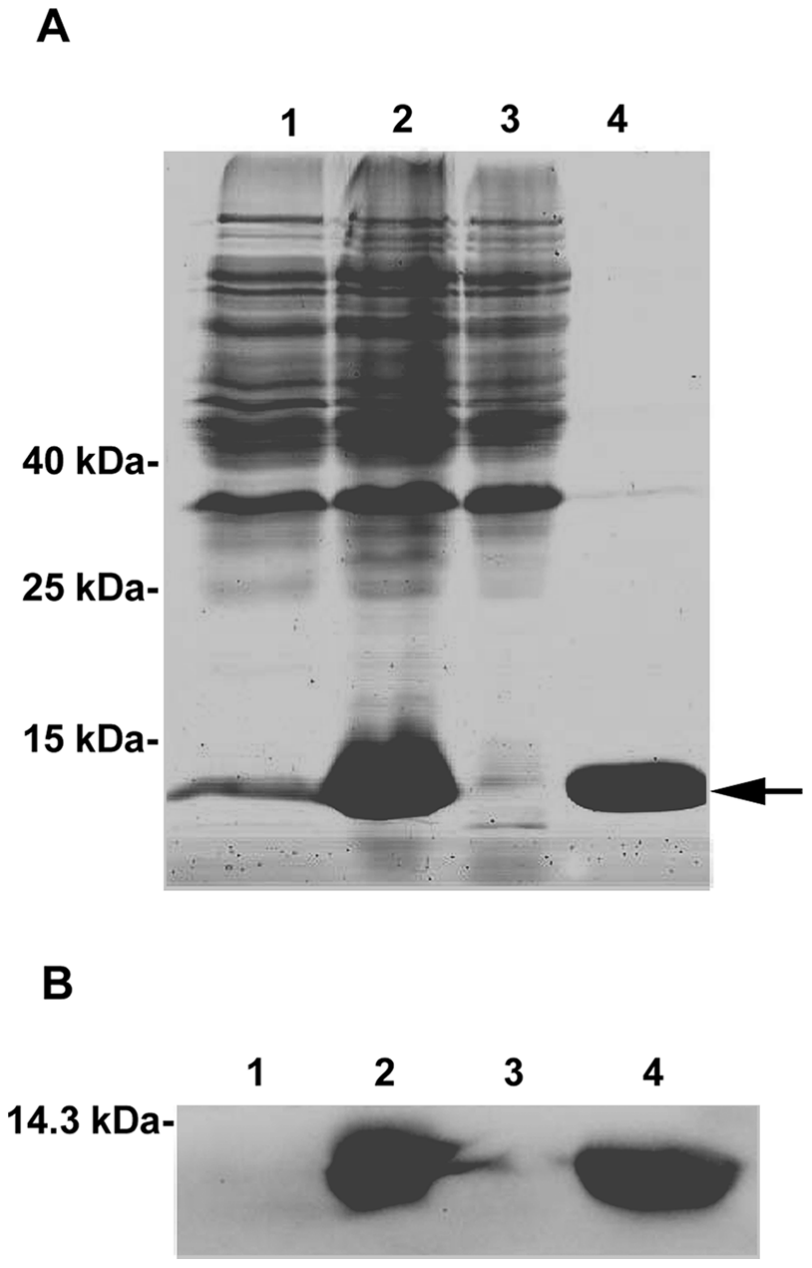
Expression and purification of the rCcTrx1 fusion protein. (A) 12% SDS-PAGE analysis of the samples collected from different steps of expression and purification. Lane 1, whole cell lysates of recombinant *E. coli* BL21 (DE3) before induction; lane 2, whole cell lysates of recombinant *E. Coli* BL21 (DE3) after induction with 1 mM IPTG for 8 h at 25°C; lane 3, fractions from the 30 mM imidazole wash of the HisTrap HP affinity column; lane 4, fractions from the 500 mM imidazole elution of the HisTrap HP affinity column. The position corresponding to the rCCTrx1 protein is indicated by an arrow. (B) Western blotting analysis of anti-His antibody cross-reactivity of the proteins separated by SDS-PAGE. The lanes are the same as described for SDS-PAGE in panel A.

Size and purity of the recombinant protein were analyzed by SDS-PAGE. As shown in Figure 5A, a single band at about 12 kDa was visualized in the fraction of the eluting peak. This band is corresponded well with the calculated size of the rCcTrx1 protein (11.5 kDa of CcTrx1 peptide with the 1.1 kDa of His-tag). Subsequently, the rCcTrx1 fusion protein was confirmed via western blotting analysis using anti-His antibodies (Figure 5B). Therefore, it was evident that the His-tagged rCcTrx1 protein was successfully expressed and purified to a high level.

### Disulfide reductase activity of the rCcTrx1 protein

**Figure 6:**
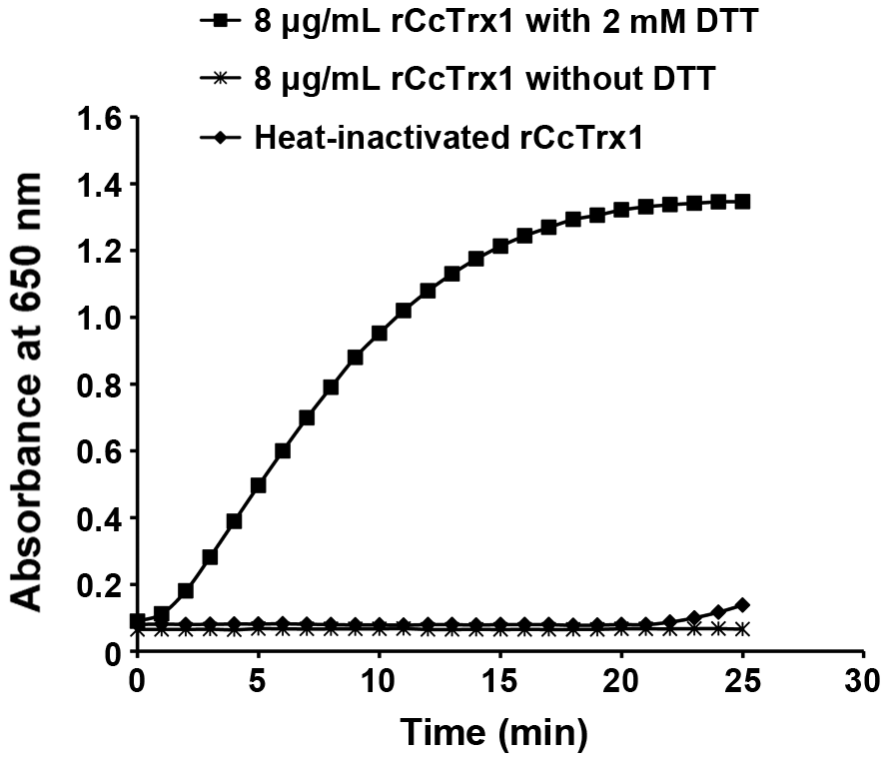
Disulfide reductase activity of the rCcTrx1 protein. Insulin disulfide reduction activity of the rCcTrx1 protein was measured in the presence of 2

Trx has been reported to function as a reductive factor through its dithiol group. In this study, insulin disulfide reduction assay was employed to investigate the dithiol-reducing enzymatic activity of the rCcTrx1 protein. The result showed that the rCcTrx1 protein distinctly displayed a specific activity to reduce insulin disulfides in a time-dependent manner and insulin reduction was rapidly increased almost from the beginning of incubation (Figure 6). However, no significant changes were observed in the control groups. In addition, the specific activity of rCcTrx1 protein was calculated to be 9.22 according to the method previously described [33]. It was comparable to the specific activity of Trx proteins from shrimp *L. vannamei* (10.44), *E. coli* (4.93), calf thymus (6.50) and liver (5.09) [33], [39], but much larger than that of the mitochondrial Trx2 from abalone (1.83) [15]. Therefore, these results indicated that the CcTrx1 protein could act as an effective disulfide reductase in *C. capillata*.

### Protection of supercoiled plasmid DNA from oxidative damage

**Figure 7:**
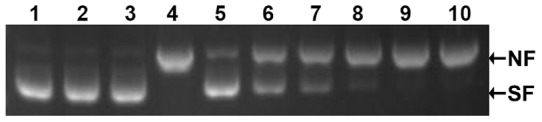
Effects of the CcTrx1 protein to protect pET-24a plasmid DNA against oxidative damage. Lane 1, pET-24a plasmid DNA only; lane 2, pET-24a plasmid DNA and 35 µM FeCl_3_; lane 3, pET-24a plasmid DNA and 10 mM DTT; lane 4, pET-24a plasmid DNA, 35 µM FeCl_3_ and 10 mM DTT; lane 5–10, pET-24a plasmid DNA, 35 µM FeCl_3_, 10 mM DTT and different concentrations of the purified rCcTrx1 protein (150, 100, 75, 50, 25 and 10 µg/mL, respectively). The bands corresponding to the nicked form (NF) and supercoiled form (SF) are indicated on the right side.

The MCO assay was performed to evaluate the ability of the rCcTrx1 protein in protecting the supercoiled plasmid DNA from oxidative damage according to the method previously described [44], [45]. As shown in Figure 7, supercoiled DNA separately incubated with FeCl_3_ or DTT was not damaged, while the DNA incubated with both FeCl_3_ and DTT was apparently converted from the supercoiled form to a nicked one. Moreover, the addition of rCcTrx1 to the MCO system effectively decreased the amount of the nicked form of plasmid DNA in a dose-dependent manner. This result suggested that the rCcTrx1 protein can protect DNA from cleavage caused by MCO system.

## Discussion

As a representative abundant marine zooplankton, jellyfish spends nearly all of its life in direct exposure to strong sunlight, UV radiation and many xenobiotics, which may lead to an increase in the production of ROS. The defense system of the cell, composed of enzymatic and non-enzymatic antioxidants, can minimize the deleterious effects of the free radicals [46], [47]. However, until now, few research has been done to identify the antioxidant system and the representative antioxidants in jellyfish, which is essential for understanding the impact of their exposure to the harsh environment.

Previously, we have cloned and characterized a peroxiredoxin 4 homologue (CcPrx4) from a cDNA library of the tentacle of *C. capillata*. CcPrx4 showed the ability to reduce hydrogen peroxide and protect supercoiled DNA from oxidative damage [6]. In the present study, we have further cloned and characterized another important antioxidant, thioredoxin, from the same jellyfish species. Peroxiredoxin and thioredoxin are both vital antioxidants in cells. However, they still have their own structural features and respective functions. The most important function of peroxiredoxin is considered to act as a principal enzyme to regulate the intracellular H_2_O_2_ concentration and scavenge ROS [48], while thioredoxin plays an important role in oxidative defense through its disulfide reductase activity [49], [50].

CcTrx1 possesses a 479 bp ORF encoding a protein of 11.5 kDa in accordance with the molecular mass of Trx1 as previously described [51], [52]. Sequence analysis revealed that the deduced amino acid sequence of CcTrx1 protein shared a significant homology with Trx1 proteins from various species and also contained the Trx structurally important characteristic CGPC active site. This active site is highly conserved in all the Trx proteins and has been demonstrated to be ideally suited to control protein function via regulation of the redox state of structural or catalytic thiol groups [11]. In addition, no signal peptide was predicted within CcTrx1, indicating that the protein encoded by the isolated gene sequence might be a cytosolic form. Thus on the basis of these typical characteristics, CcTrx1 was proposed to be a new member of the Trx1 family.

Structural analysis of Trx proteins from fruit fly *Drosophila melanogaster* and human suggested that dimerization could occur in Trx, which would block the active site [40]. In human, the Trx dimer was linked through a disulfide bond between Cys73 of each monomer [41]. However, the absence of Cys73 in the CcTrx1 protein, which is also revealed in the Trx1 proteins from *H. magnipapillata, R. philippinarum*, *N. scintillans*, *H. vulgaris*, *N. vectensis* and *A. queenslandica*, suggests that such disulfide bond formation would not occur in this molecule. However, a similar dimer may still be present in the CcTrx1 protein with the corresponding residue alanine which is also reported in human mitochondrial Trx2 [53]. It has been reported that the fold of Trx protein consists of five β-strands surrounded by four α-helices [11]. The β-strands and α-helices can be divided in an N-terminal β1α1β2α2β3 and a C-terminal β4β5α4 motif connected by the α3-helix. The catalytic CGPC motif is located after the β2-strand and at the amino-end of the α2-helix. We found that the predicted three-dimensional structure of the CcTrx1 protein also had this characteristic structure of Trx fold, which had been reported to exist in many critical enzymes in the thiol-dependent antioxidant system, such as glutaredoxin, peroxiredoxin, and glutathione peroxidase [54]–[56].

In the pairwise comparisons analysis, the CcTrx1 protein was found to have a maximum similarity with that of *R. philippinarum* rather than that of other cnidarians we previously expected. This result was further confirmed by the phylogenetic tree analysis, which showed that the CcTrx1 protein was placed closer to that of *R. philippinarum* than those of other cnidarians including *H. magnipapillata, H. vulgaris* and *N. vectensis*.

Tissue-specific expression analysis showed that CcTrx1 was constitutively expressed in all the tested jellyfish tissues including tentacle, umbrella, oral arm and gonad. Its wide distribution may indicate its participation in many important physiological functions in *C. capillata*. In addition, the higher expression levels of CcTrx1 transcripts were found in the tentacle and umbrella, suggesting that these tissues might be the main metabolic centers for ROS production in jellyfish.

To obtain the rCcTrx1 fusion protein, we constructed the recombinant plasmid CcTrx1/pET-24a, and transformed it into *E. coli* BL21 (DE3). SDS-PAGE and Western blotting results demonstrated that the CcTrx1 protein was successfully expressed and purified to a high level. Trx has been demonstrated to participate in many essential antioxidant and redox-regulatory processes via a pair of conserved cysteine residues [57]. In this study, insulin disulfide reduction assay was further performed to evaluate the enzymatic redox activity of the rCcTrx1 protein. The result suggested that this protein was able to catalyze the insulin disulfide bond reduction in the presence of DTT. Therefore, it is proposed that different proteins with disulfide bonds may be the potential substrates for CcTrx1 protein, which awaits further functional confirmation. These results indicate that the rCcTrx1 protein is biologically active and probably plays an important role in the physiological reduction of disulfide bonds and in the regulation of protein metabolism.

Trx has been demonstrated to possess a general intracellular antioxidant activity and protection against oxidative stress [58], [59]. In the present study, the antioxidant capacity of the rCcTrx1 protein was also evaluated. MCO system can generate ROS, which is responsible for DNA damage described as nicking of supercoiled DNA [60]. MCO assay results confirmed that the rCcTrx1 protein had the ability to detoxify the ROS produced by the MCO system and protect supercoiled pET-24a plasmid DNA from oxidative damage in a concentration-dependent manner. These results conformed to the illustration that Trx might play an important role the in defense against oxidative stress. Therefore, CcTrx1 may be a functional homologue of Trx1, which represents a potential protective barrier against oxidative damage in the body of *C. capillata*.

## Conclusions

We identified and characterized a full-length cDNA with homology to Trx family of proteins, CcTrx1, from the jellyfish *C. capillata*. CcTrx1 mRNA was constitutively expressed in the tested tissues. The rCcTrx1 protein was demonstrated to possess the expected redox activity in enzymatic analysis and protect supercoiled DNA from oxidative damage. Our results suggest that CcTrx1 may function as an important antioxidant and protect *C. capillata* against oxidative stress. To the best of our knowledge, very little is known about oxidative stress processes in cnidarians, and this is the first report of a full-length Trx gene isolated and characterized from a marine cnidarian, which enriches our understandings of Trx proteins and provides scientific foundations for the expression pattern and regulatory role of Trx protein in cnidarians.
